# The spatial distribution, accumulation and potential source of seldom monitored trace elements in sediments of Three Gorges Reservoir, China

**DOI:** 10.1038/srep16170

**Published:** 2015-11-05

**Authors:** Lanfang Han, Bo Gao, Huaidong Zhou, Dongyu Xu, Xin Wei, Li Gao

**Affiliations:** 1 State Key Laboratory of Simulation and Regulation of Water Cycle in River Basin, China Institute of Water Resources and Hydropower Research, Beijing 100038, China; 2 Department of Water Environment, China Institute of Water Resources and Hydropower Research, Beijing 100038, China; 3 State Key Laboratory of Water Environment Simulation, School of Environment, Beijing Normal University, Beijing 100875, China

## Abstract

The alteration of hydrologic condition of Three Gorges Reservoir (TGR) after impoundment has caused numerous environmental changes. This study investigated the distribution, accumulation and potential sources of the seldom monitored trace elements (SMTEs) in sediments from three tributaries (ZY, MX and CT) and one mainstream (CJ) in TGR during different seasons. The average contents of most SMTEs excluding Sb in the winter were similar to that in the summer. For Sb, its average concentrations in the summer and winter were roughly six and three times higher than its background value, respectively. Contamination factor (CF) and geoaccumulation index (*I*_geo_) demonstrated that most of the sediments were obviously contaminated by Sb. The enrichment factors (EF) of Ga and Sb were higher than 2.0, revealing the possible anthropogenic inputs; However, the EFs of other SMTEs were lower than 1.5, indicating the natural inputs. Correlation and principal component analysis suggested the most SMTEs were positively correlated with major elements (Cr, Mn, Cu, Zn, As, Cd and Pb) and clay contents, which implies that SMTEs had the same sources with these major metals, and the fine particles might be a major carrier for transporting SMTEs from the rivers to the TGR.

The contamination of aquatic environment by commonly monitored trace elements (e.g. Cu, Hg, Pb, As, Cd, Cr, Mn, and Zn) has been in focus for decades^1^,^2^,^3^. Comparatively, the so-called “seldom monitored trace elements (SMTEs)”, such as Ga, Sn, Sb, Be, Tl, Bi and Li, have attracted rather less attention. The SMTEs can be introduced into the rivers by natural processes including erosion of ore-bearing rocks, wind-blown dust, volcanic activity and forest fires, and processes derived from human activities via atmospheric deposition, direct discharges or dumping^4^. Coal and fossil fuel combustion and steel manufacturing plants are the primary anthropogenic sources of SMTEs^5^. Many SMTEs are deemed serious contaminants due to their toxicity, persistence and non-degradability in the environment. For instance, Sb, Tl and their compounds are classified as priority contaminants by the European Union and the Environmental Protection Agency of the United States and are found on the list of banned hazardous compounds specified in the Basel Convention^6^,^7^. The anthropogenic release of Sb and Tl into the environment is significant. In the last decades, global fluxes of Sb have increased at least 10 fold, resulting in an increase of environmental Sb contamination^8^. In, Ti and Bi are also known to be non-essential and pose harm to living organisms^9^,^10^.

Once entered the aquatic environment, SMTEs would precipitate into bottom sediments or be adsorbed on sediment particles. Because of their relatively low abundance, SMTEs are particularly sensitive to surrounding environmental conditions. It has been identified that the concentrations of SMTEs in sediments remarkably varied with the physical, chemical and biological processes of aquatic environment^11^. The high potential of sediments to accumulate compounds makes them a major repository of natural and anthropogenic SMTEs and one of the most important tools to evaluate the contamination level of aquatic ecosystems^5^. Consequently, the knowledge of the concentrations and distributions of SMTEs in sediments plays a crucial role in detecting sources of SMTEs and assessing the ecological risks of SMTEs in aquatic systems.

The Three Gorges Reservoir (TGR) region is one of several huge projects in China that are transforming the Chinese environment^12^. It covers an area of 58,000 km^2^ that includes 19 counties and cities where 1.13 million people will eventually be resettled^13^. It sustains the health of the ecological environment in the middle and lower reaches of the Yangtze River, and the water supply security of the whole nation. However, TGR is very prone to receive significant metal inputs from shipping activities, industrial, agricultural and domestic discharges from the nearby cities. As a result, the contamination status of the aquatic environment in the TGR should be of global interest and be held of much account. Tang *et al.*^14^ reported that the impoundment of the TGR has significantly altered the hydrologic regime within the dammed reaches. Nevertheless, after impounding water into the TGR, little concern has arisen over on the behaviors of SMTEs in the TGR sediments. Therefore, the objectives of this study were: 1) to investigate the SMTEs concentration and spatial distribution in the TGR sediments during two different impounding periods; 2) to evaluate the enrichments of SMTEs in the TGR sediments; 3) to finally discuss the possible origins of SMTEs.

## Results and Discussion

### Concentrations of SMTEs in surface sediments

As shown in Fig. S1, the TGR sediments sampled in the summer and winter were dominated by silt, with a small portion of sand. On average, clay, silt and sand accounted for 25.4%, 70.1% and 4.5% of the sediments, respectively. According to Shepard’s classification^15^, there were two main types of sediment, namely silty clay and clay, in this region (Fig. S1). Table 1 summarizes the means, ranges and standard deviation (S.D.) of SMTEs concentrations in the surface sediments of all studied stations in the TGR. There was a wide range in concentrations for the different SMTEs (3–4 orders of magnitude). Based on the data in Table 1, Tl and Bi could be described as typically low concentration elements (0.30–1.10 mg/kg). An intermediate group was formed by Be, Co, Sn and Sb (1–25 mg/kg), whereas V was a group with much higher concentration (>90 mg/kg). As shown in Table 1, the Li, Be, B, V, Co, Ni, Ga Sn, Sb, Tl and Bi concentrations in the surface sediments sampled in the summer were in the range of 43.61–77.09, 2.00–3.43, 68.46–127.24, 90.36–212.05, 13.51–22.70, 29.31–59.71, 20.17–61.78, 2.51–4.45, 3.16–8.45, 0.55–1.05 and 0.36–0.81 mg/kg, with averages of 61.56, 2.82, 93.85, 143.77, 18.54, 46.13, 37.09, 3.51, 5.53, 0.75 and 0.56 mg/kg, respectively. The mean contents of most SMTEs excluding Sb in the winter were similar to that in the summer (Table 1). The mean Sb concentration in the summer was roughly 1.6 times higher than that in the winter. Application of the K–S test confirmed that the concentrations of most of SMTEs (except B in the summer and Sb in the winter) were normally distributed (Table 1). After log-transformation, the distributions of B and Sb were normally distributed. All SMTEs in this study were higher than the local background values (Table 1), which likely results from the impoundment of TGR. After impoundment, the average water flow velocity decreased^16^, which could in turn accelerate the sinking process of the suspended particular matters and the metals attached to these particles. In particular for Sb, its mean concentrations in the summer and winter were roughly six and three times higher than its background value, respectively, suggesting the severe contamination of Sb in this area. The large number of mining activities and metal smelting processes in the nearby cities of TGR may be possibly responsible for the serious Sb contamination^17^.

### Spatial distribution of SMTEs in sediments

**Figure 1:**
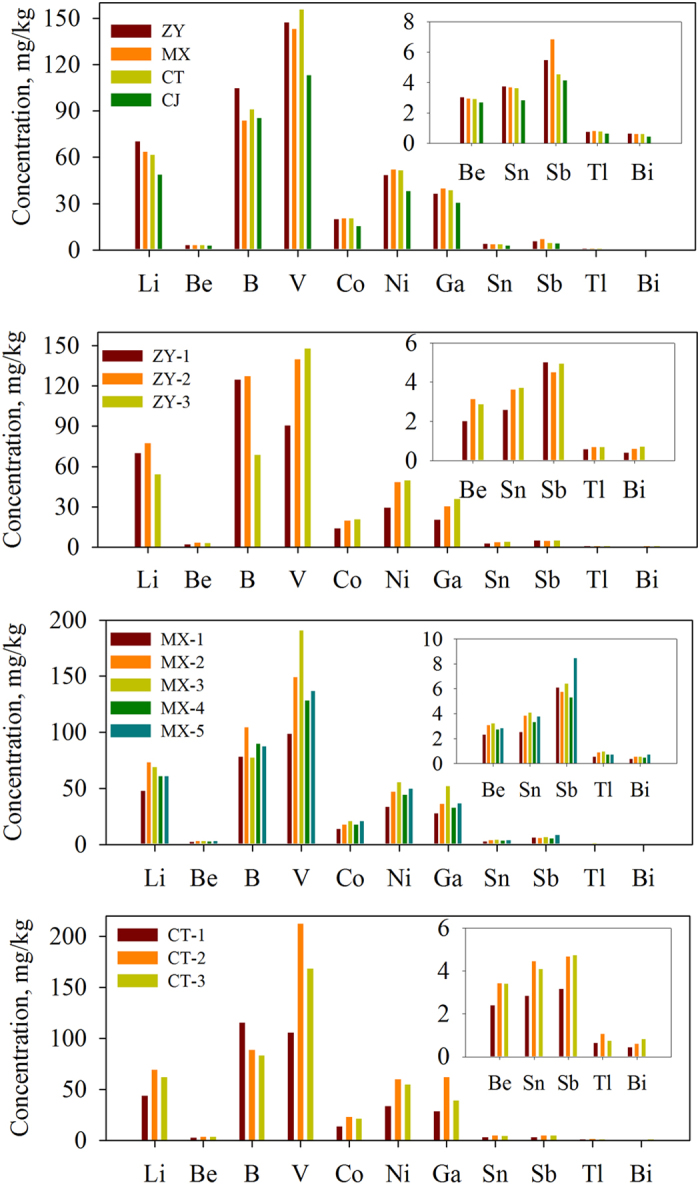
The comparison of seldom monitored trace elements (SMTEs) concentrations between mainstream and tributaries in the summer, and SMTEs concentration distributions in each tributary—ZY, MX and CT.

The spatial distribution of SMTEs in the TGR sediments is displayed in Fig. 1 and Fig. S2. The SMTEs concentrations did not show the regular distribution among the sediments in three tributaries (ZY, MX and CT) and mainstream (CJ) during two reasons. For example, as listed in Fig. 1a, the peak of mean Li, Be, B, Sn and Bi concentrations in the summer were in ZY, while Sb and Tl were in MX, Co and Ni were in CT, V and Ga were in CJ. The concentrations of V, Co, Ni, Ga, Sn and Bi in the summer and Tl and Li in the winter rose from upstream to downstream of ZY, while the similar pattern did not occur in other tributaries (Fig. 1 and Fig. S2). With respect to CT, the concentrations of some SMTEs (e.g. Li, Be, V, Ni, Ga and Tl) in midstream (CT-2) were higher than that in upstream (CT-1) and downstream (CT-3) (Fig. 1d). The change of hydraulic conditions due to the impoundment of TGR likely accounts for the higher metal concentrations in midstream. With the success of second-phase water storage project for the TGR, the water from mainstream and upstream of tributary converges in the midstream of tributary. This may lead to that the aquatic environment of the midstream of tributaries changes from typical river water to a kind of water body like lake. The slow water flow under this condition could result in the sedimentation of more suspended particles and attached metal into the sediments.

**Figure 2:**
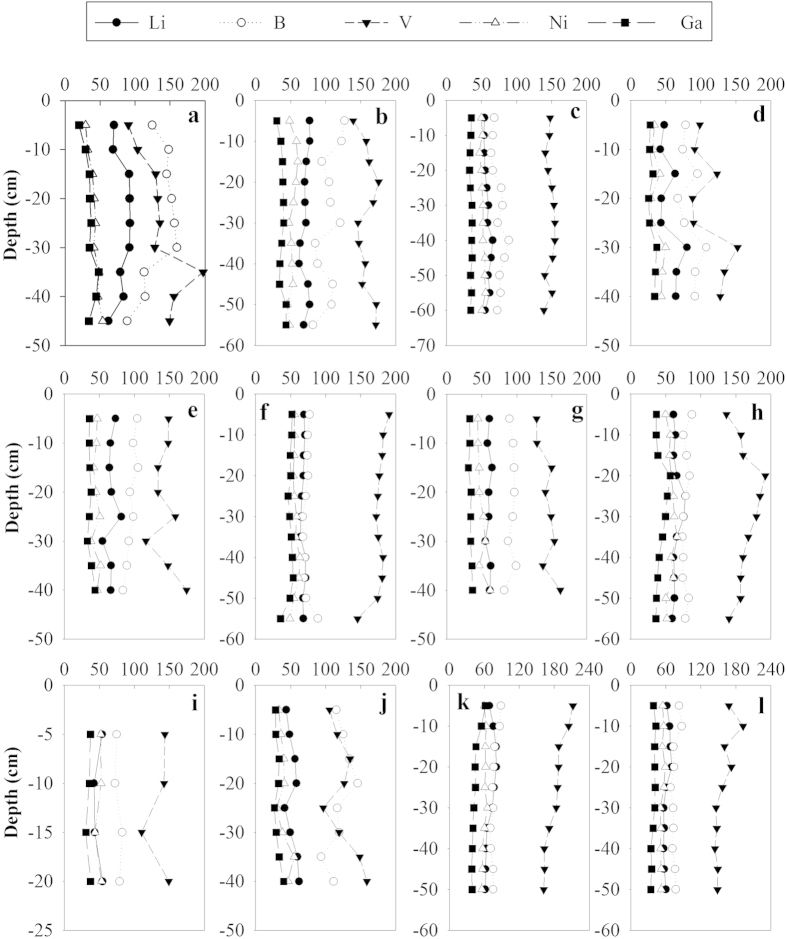
The distribution of concentrations of Li, B, V, Ni and Ga with the sediment profiles in the mainstream (CJ) and tributaries (ZY, MX and CT) of the Three Gorges Reservoir during the summer. Note a = ZY-1, b = ZY-2, c = ZY-3, d = MX-1, e = MX-2, f = MX-3, g = MX-4, h = MX-5, i = CJ, j = CT-1, k = CT-2, l = CT-3.

Among the all SMTEs, Ni, Ga, Be, Sn, Tl and Bi in the summer and Ni, Ga, Be, Co, Tl and Bi in the winter had the similar vertical profiles (Fig. 2 and Fig. S3–S5), showing an insignificant variation with the increasing sediment depth. This implies a small anthropogenic contribution to these metals. However, B, V, Co and Sb in the summer and B, V, Sn and Sb in the winter had the different trends (Fig. S3–S5). In general, they displayed a fluctuant change with the depth. For example, the elevated V concentration in ZY-1 in the summer was observed in the upper 35 cm (Fig. 2a), thereafter the concentrations decreased. The concentrations of all SMTEs consistently reached peak values at 30 cm in the MX-1, with the concentrations of 80.19 mg/kg for Li, 3.23 mg/kg for Be, 107.76 mg/kg for B, 152.44 mg/kg for V, 19.18 mg/kg for Co, 49.53 mg/kg for Ni, 36.97 mg/kg for Ga, 3.54 mg/kg for Sn, 4.14 mg/kg for Sb, 0.81 mg/kg for Tl, 0.57 mg/kg for Bi (Fig. 2d). In addition, the fluctuations of B and Co in the summer and B and Sn in the winter with the sediment depth in the downstream of each tributary were less remarkable than that in the upstream (Fig. 2 and Fig. S3–S5).

### Assessment of SMTEs contamination in sediments

The degree of contamination from SMTEs was assessed by determining the contamination factor (CF), geoaccumulation index (*I*_geo_) and enrichment factor (EF). CF, the ratio of the measured concentration to natural abundance of a given metal, could be classified into four grades for monitoring the pollution of one single metal over a period of time^18^: low degree (CF < 1), moderate degree (1 ≤ CF < 3), considerable degree (3 ≤ CF < 6), and very high degree (CF ≥ 6).

**Figure 3:**
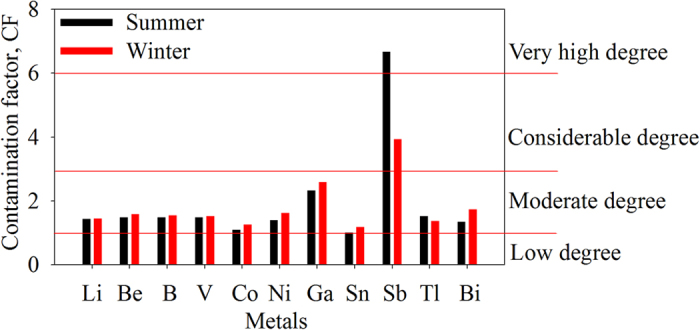
Contamination factor (CF) of seldom monitored trace elements in surface sediments of the Three Gorges Reservoir during summer and winter.

The value of contamination factor (CF) for all metals showed moderate degree of contamination (CF > 1), whereas Sb in the sediments sampled in summer showed very high degree of contamination (CF > 6) (Fig. 3). The mean CF values of Li, Be, B, V, Co, Ni, Ga, Sn, Sb, Tl and Bi were 1.43, 1.48, 1.49, 1.09, 1.40, 2.32, 1.00, 6.66, 1.53 and 1.34 during summer and 1.45, 1.59, 1.55, 1.53, 1.26, 1.62, 2.58, 1.18, 3.93, 1.38 and 1.73 during winter season.

*I*_geo_ introduced by Müller^19^ is an important geochemical criterion to evaluate metal contamination in sediments and has been used since the late 1960s. In this study, the *I*_geo_ for the TGR sediments was calculated using Eq. (1).


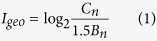


where *C*_*n*_ is the measured concentration of trace metal (*n*) in the sediment, *B*_*n*_ is the geochemical background value of element *n* in the sediment^20^, and 1.5 is the background matrix correction factor due to lithogenic effects. Seven classes of the *I*_geo_ were adopted: uncontaminated (<0), uncontaminated to moderately contaminated (0–1), moderately contaminated (1–2), moderately to strongly contaminated (2–3), strongly contaminated (3–4), strongly to extremely contaminated (4–5), and extremely contaminated (>5).

The results of the calculated *I*_geo_ values with respect to each SMTE are listed in Table 2. In general, the average *I*_geo_ values in the summer and winter are −0.09 and −0.05 for Li, −0.04 and 0.07 for Be, −0.04 and 0.02 for B, −0.07 and 0.01 for V, −0.49 and −0.26 for Co, −0.14 and 0.09 for Ni, 0.55 and 0.78 for Ga, −0.61 and −0.35 for Sn, 2.09 and 1.38 for Sb, −0.01 and −0.13 for Tl, −0.21 and 0.19 for Bi (Table 2). The average *I*_geo_ values in the summer and winter were in the order: Sb > Ga > Tl > B > Be > V > Li > Ni > Bi > Co > Sn and Sb > Ga > Bi > Ni > Be > B > V > Li > Tl > Co > Sn, respectively. Sb had the highest average *I*_geo_ values among all the SMTEs and was classified as ‘moderately to strongly’ level in the summer and ‘moderately contaminated’ in the winter (Table 2). This indicates that Sb could be the dominated contaminants in the TGR sediments. Similarly, Gao *et al.*^21^ documented that the *I*_geo_ of Sb was the highest among the metals in the sediments of Beijiang River. Duan *et al.*^5^ also found that Sb was one of the most anthropogenic enriched elements in the surface sediment of Bohai Bay. For the three tributaries, MX was extremely strongly contaminated for Sb. In contrast, the average *I*_geo_ values of Li, Co, Sn and Tl during two reasons were consistently less than zero (Table 2), suggesting that TGR sediments were uncontaminated with respect to these SMTEs.

Similar to *I*_geo_, EFs can be utilized as a reference to predict the extent of metal contamination^22^. The EFs for SMTEs in all the sediments samples were calculated according to the following formula:


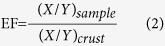


where (*X/Y)*_sample_ is the ratio of element (X) to normalizer element (Y) for the studied samples and (*X/Y*)_crust_ is the ratio of element (X) to normalizer element (Y) for earth crust. In the literature, some conservative elements such as Al^23^, Li^24^, Cs^25^, Sc^26^, V, Co, Ni^27^, Fe^22^ and even organic matter content^28^ had been employed as reference elements. In this study, Co displayed the significant relationships with almost all SMTEs (Table S1 and S2). Moreover, the mean concentration of Co was just 1.2 times higher than local background value (Table 1), implying the natural origin. Matthai and Birch^29^ have proved that Co is a suitable normalizing element in the area for which there is a demonstrated absence of substantial anthropogenic origin of this trace metal. Hence, Co was employed as the reference elements. It is generally accepted that EF <2 reflects natural variability of the sample mineralogical composition^30^,^31^; beyond 2, a significant enrichment is suspected. Based on the research of Zhang and Liu^32^, if an EF value is between 0.5 and 1.5, it suggests that SMTEs may be entirely from crustal materials or natural weathering processes. If an EF value is greater than 1.5, it indicates that a significant portion of SMTEs are provided by other sources rather than crustal or natural origins.

The average EF values in Table 3 showed that the mean EFs values of Sb and Ga in wet and dry reasons were higher than 1.5, while that of other SMTEs remained within the range of natural variability. The EFs of these SMTEs in the summer and winter lied in the following sequences: Sb > Ga > B > Tl > Be > Li > V > Ni > Bi > Sn; Sb > Ga > Bi > B > Ni > Be > V > Li > Tl > Sn (Table 3). Thus, Sb and Ga were the most anthropogenic enriched elements in the TGR sediments. Except for Sb and Ga, the EF values of other SMTEs were close to or lower than one, revealing that they were derived from crust. Furthermore, Sutherland^31^ proposed that EF values between 5 and 20 suggesting a significant enrichment. In this respect, the sediments from ZY-1, MX-1, MX-2, MX-3, MX-4, MX-5 and CJ in the summer were significantly contaminated by Sb (Table 3).

### Potential Origins of SMTEs

Seven commonly monitored metals (Cr, Mn, Cu, Zn, As, Cd and Pb) were also measured in our study (Table S3). Their mean concentration ranges were as follows: 51.74–131.08, 218.23–766.71, 22.934–124.44, 64.80–271.24, 7.35–20.82, 0.71–2.52 and 19.03–121.73 mg/kg, with averages of 100.26, 550.32, 58.47, 141.23, 14.55, 0.84 and 43.07 mg/kg, respectively (Table S3). The trace metals could be introduced in the environment from both natural and anthropogenic activities. Atmosphere deposition, riverine input and food chain transportation were identified to be the major pathways of SMTEs in the aquatic environment^5^. Yi *et al.*^33^ identified that Cd, Pb, Cr, Cu, and Zn in sediments in the middle and lower reaches of the Yangtze River primarily came from the riverine inputs and atmospheric deposition. Wang *et al.*^34^ pointed out that atmospheric deposition and stormwater runoff significantly contributed to the Pb and Zn contamination in Yangtze river sediments. Thus, riverine inputs and atmospheric deposition may be the two important anthropogenic sources (or “input pathways”) of these major metals in the TGR sediments. The quantitative analyses of the possible relationships were carried out among seven major elements and SMTEs, and the data obtained for two seasons are presented in Tables S1 and S2. The results indicated that the seven metals were strikingly and positively related to the concentrations of most SMTEs (except Li, B, and Tl in the summer and Li, Be, B, Sb and Tl in the winter), suggesting that they had the same sources, and the factors controlling major elements distributions also worked on the SMTEs. Therefore, the riverine input and atmospheric deposition may also be the main input pathways of these SMTEs. Different from the study by Duan *et al.*^5^, no positive/negative relationship occurred between concentration of each SMTE and total organic carbon (TOC) content (Tables S1 and S2), reflecting that organic matter may not be the major sink of SMTEs in this study. However, it was found that Be, V, Co, Ni, Ga, Sn, Sb and Bi during the summer and Be, V, Co, Ni, Sn, and Bi during the winter were positively associated with Mn, implying that Mn-oxides had high affinity for these SMTE contaminants (Table S1 and S2). Additionally, correlation analysis suggested that SMTEs were significantly correlated with clay contents (Table S1 and S2), indicating that the SMTES were mainly combined with the fine particles. Hence, the fine particles might be a major carrier for transporting SMTEs from the rivers to the TGR. So, according to the distribution of fine-grained sediment, the physical transportation of sediments and associated SMTEs could be reflected.

**Figure 4:**
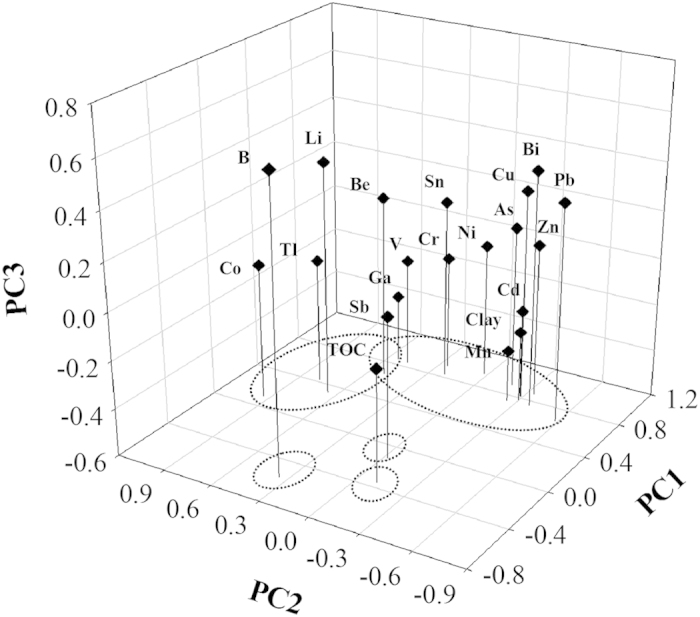
Plot of the first three principal components accounting for the 76.75% of variance.

A principal component analysis (PCA) was performed on selected data of the sediments for the two seasons to reveal the interrelationships of SMTEs and the major constituents (Cr, Mn, Cu, Zn, As, Cd, Pb, TOC and clay) (Table 4 and Fig. 4). Four principal components (PC) with eigenvalues higher than 1 were extracted. The graphic representation of the first three PC is also shown in Fig. 4 (PC4 involves only one metal), in where the associations between metals can be seen. As listed in Table 4, PCA leaded to a reduction of the initial dimension of the dataset to four components which explain a 82.3% of the data variation. Individually, first PC (PC1), which has the high loadings of V, Ni, Ga, Sn, Cr, Cu, Zn, As and Cd, and medium loading of Be, Bi, Mn, Pb and clay and accounts for 55.5% of variance, appears to represent an ‘anthropogenic factor’; Li, Be, Co, Ga and Tl were mainly associated with PC2, and explains 15.2% of variance; B formed a third group; PC4 has high loading of Sb only and can be considered as a Sb contamination factor (Table 4), which coincides with the above discussion that Sb concentration was exceptionally higher than other SMTEs.

## Materials and Methods

### Sampling sites

**Figure 5:**
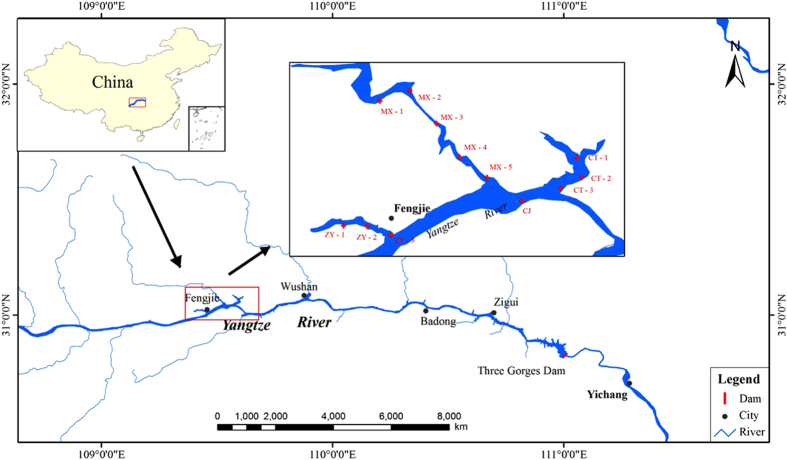
Sampling sites in the Three Gorges Reservoir. The map was generated using the software of ArcGIS 9.0.

Twelve sediment cores were collected from three tributaries (Zhuyi river (ZY), Caotang river (CT) and Meixi River (MX)) and one mainstream (CJ) in the lower reaches of the TGR in July 2014 (submerged by impoundment in 2008), and six sediments cores were from two tributaries (ZY and CT) in November 2014. The information about the sampling sites is described in Fig. 5 and this map was generated using the software of ArcGIS 9.0. At each sampling site, sediment core samples were taken using a core sampler (K-B type, Wildco, USA) near the middle of the flow of the stream. Each of the sediment cores was cut into 5-cm length sections to obtain subsamples. The sediment samples were placed in clean polyethylene bags and treated immediately on returning to the laboratory. The sediment samples were wet sieved through an acid-cleaned 63-μm mesh nylon sieve in order to obtain the chemically active material, dried at 40 °C to constant weight, and ground in an agate mortar to ensure homogeneity.

### Analytical methods

All chemical treatments were in the ultra-clean laboratory, and all reagents were high purity grade. Total metal concentrations in the sediments were measured using established methods^35^. Briefly, a mass of 40 mg of dry sample was weighed and dissolved into 10 mL Teflon bombs. About 2 mL concentrated HNO_3_ + 0.2 mL concentrated H_2_O_2_ were added to samples and was left on a hot plate for one day. This step was to remove organic materials from sediment samples. The samples were then dried at 120 °C. The residue was dissolved in 1 mL HNO_3_ + 1 mL HF of sample. After 30 min ultrasonic procedure, the samples were taken into sealed bombs and were placed in an oven at 190 °C for 48 h. This procedure resulted in clear solutions for sediment sample. After evaporation at 120 °C, samples were dissolved in 1% HNO_3_. Inductively coupled plasma-mass spectrometry (ICP-MS, Perkin Elmer Elan DRC-e) was used to determine the total concentrations of SMTEs (Li, Be, B, V, Co, Ni, Ga, Sn, Sb, Tl and Bi) and Cr, Cu, Zn, Cd and Pb. Mn and As. The quality controls for the strong acid digestion method included reagent blanks, duplicate samples, and standard reference materials. The QA/QC results show no sign of contamination in all the analysis. The accuracy of the analytical procedures employed for the analysis of the trace elements in sediments was checked using the certified reference material of stream sediment (GSD-12, GBW07312), obtaining good agreement with the certified values (Table S4).

### Grain size and total organic carbon analysis

The granularity of each sample was analyzed using a particle size analyzer (Mastersizer 2000; Malvern, United Kingdom) with the ability to analyze a particle size range of 0.02–2000 μm. The particle size ranges used were <4 μm (clay), 4–63 μm (silt), and >63 μm (sand). TOC in decarbonated sediments was analyzed using an Elementar Vario MACRO Cube CHNS analyzer.

### Statistical analysis

Statistical methods including Pearson correlation analysis and PCA were used to elucidate the relationships among SMTEs. In this work, a value of *p* < 0.05 was considered to indicate a significant difference in all statistical analysis. Pearson correlation analysis had been used to extract information from the chemical analysis in order to find the relationships between SMTEs and major elements, TOC and clay contents. PCA was executed on the analytical data in order to obtain a visual representation of the main characteristics of the relationships among SMTEs, major elements, TOC and clay contents. Usually the PCs were obtained by their eigenvalues > 1. All statistical analyses were performed using the SPSS 16.0.

## Additional Information

**How to cite this article**: Han, L. *et al.* The spatial distribution, accumulation and potential source of seldom monitored trace elements in sediments of Three Gorges Reservoir, China. *Sci. Rep.* **5**, 16170; doi: 10.1038/srep16170 (2015).

## Supplementary Material

Supporting Information

## Figures and Tables

**Table 1:** Seldom monitored trace elements concentrations in surface sediments of the Three Gorges Reservoir (mg/kg) during summer (July) and winter (November).

Sampling sites	Li	Be	B	V	Co	Ni	Ga	Sn	Sb	Tl	Bi
**Summer (July)**											
ZY-1	69.97	2.00	124.50	90.36	13.94	29.31	20.17	2.58	4.99	0.58	0.39
ZY-2	77.09	3.13	127.24	139.58	19.58	48.37	30.19	3.62	4.51	0.69	0.58
ZY-3	54.20	2.87	68.46	147.92	20.76	49.72	35.54	3.69	4.92	0.67	0.70
MX-1	47.68	2.31	78.30	98.61	13.88	33.53	27.54	2.51	6.08	0.55	0.36
MX-2	73.16	3.07	104.19	148.98	17.46	46.89	36.15	3.82	5.75	0.89	0.53
MX-3	68.92	3.21	77.23	190.71	20.90	55.55	51.90	4.06	6.40	0.97	0.54
MX-4	60.72	2.70	89.48	128.17	17.49	44.32	32.63	3.32	5.27	0.72	0.48
MX-5	60.95	2.83	87.16	136.70	20.68	49.51	36.55	3.75	8.45	0.70	0.72
CJ	54.00	2.67	75.06	143.60	21.30	51.99	37.40	3.49	6.87	0.69	0.62
CT-1	43.61	2.40	115.17	105.64	13.51	33.30	28.56	2.84	3.16	0.64	0.43
CT-2	68.92	3.43	88.42	212.05	22.70	59.71	61.78	4.45	4.68	1.05	0.59
CT-3	61.88	3.41	82.98	168.10	21.20	54.59	38.89	4.08	4.72	0.74	0.81
Minimum Value	43.61	2.00	68.46	90.36	13.51	29.31	20.17	2.51	3.16	0.55	0.36
Maximum Value	77.09	3.43	127.24	212.05	22.7	59.71	61.78	4.45	8.45	1.05	0.81
Mean (*N* = 110)	61.56	2.82	93.85	143.77	18.54	46.13	37.09	3.51	5.53	0.75	0.56
S.D. (*N* = 110)	10.36	0.44	19.86	35.85	3.28	9.62	11.07	0.61	1.35	0.15	0.14
K-S test	0.715^a^	1.209^a^	**1.610**	0.764^a^	1.302^a^	0.99^a^	1.267^a^	1.101^a^	0.12^a^	0.656^a^	0.775^a^
**Winter (November)**											
ZY-1	57.11	2.98	78.35	142.72	22.52	53.26	41.31	4.21	2.96	0.64	0.65
ZY-2	57.48	2.90	77.00	155.15	20.77	59.60	43.78	3.96	3.89	0.66	0.85
ZY-3	63.83	2.30	132.04	109.58	16.62	35.90	39.73	3.17	3.20	0.73	0.52
CT-1	60.34	3.17	105.02	157.26	22.13	54.01	39.75	4.15	2.77	0.70	0.73
CT-2	71.18	3.57	106.74	170.04	23.47	60.64	39.82	4.57	3.04	0.68	0.79
CT-3	64.26	3.18	86.59	153.68	23.32	56.41	43.70	4.78	3.72	0.64	0.82
Minimum Value	57.11	2.30	77.00	109.58	16.62	35.90	39.73	3.17	2.77	0.64	0.52
Maximum Value	71.18	3.57	132.04	170.04	23.47	60.64	43.78	4.78	3.89	0.73	0.85
Mean (N = 42)	62.37	3.02	97.62	148.07	21.47	53.3	41.35	4.14	3.26	0.67	0.73
S.D. (N = 42)	5.27	0.42	21.17	20.78	2.57	9.02	1.95	0.56	0.44	0.03	0.12
K-S test	0.569^a^	0.609^a^	0.974^a^	0.675^a^	0.797^a^	0.931^a^	1.043^a^	0.987^a^	**3.35**	1.071^a^	0.823^a^
Sediments of the Yangtze River^b^	43.00	1.90	63.00	97.00	17.00	33.00	16.00	3.50	0.83	0.49	0.42

^a^Asymptotic significance >0.05 level (two-tailed);

^b^The values were cited from Chi *et al.*^20^

**Table 2:** *I*_geo_ values of seldom monitored trace elements in surface sediments of the Three Gorges Reservoir during summer (July) and winter (November).

Sampling sites	Li	Be	B	V	Co	Ni	Ga	Sn	Sb	Tl	Bi
**Summer (July)**											
ZY-1	0.12	−0.51	0.40	−0.69	−0.87	−0.76	−0.25	−1.03	2.00	−0.35	−0.70
ZY-2	0.26	0.14	0.43	−0.06	−0.38	−0.03	0.33	−0.54	1.86	−0.10	−0.11
ZY-3	−0.25	0.01	−0.47	0.02	−0.3	0.01	0.57	−0.51	1.98	−0.14	0.16
MX-1	−0.44	−0.30	−0.27	−0.56	−0.88	−0.56	0.20	−1.07	2.29	−0.42	−0.82
MX-2	0.18	0.11	0.14	0.03	−0.55	−0.08	0.59	−0.46	2.21	0.27	−0.24
MX-3	0.10	0.17	−0.29	0.39	−0.29	0.17	1.11	−0.37	2.36	0.39	−0.23
MX-4	−0.09	−0.08	−0.08	−0.18	−0.54	−0.16	0.44	−0.66	2.08	−0.04	−0.40
MX-5	−0.08	−0.01	−0.12	−0.09	−0.30	0.00	0.61	−0.48	2.76	−0.06	0.19
CJ	−0.26	−0.10	−0.33	−0.02	−0.26	0.07	0.64	−0.59	2.46	−0.10	−0.03
CT-1	−0.56	−0.25	0.29	−0.46	−0.92	−0.57	0.25	−0.88	1.34	−0.19	−0.56
CT-2	0.10	0.27	−0.1	0.54	−0.17	0.27	1.36	−0.24	1.91	0.51	−0.10
CT-3	−0.06	0.26	−0.19	0.21	−0.27	0.14	0.70	−0.37	1.92	0.01	0.36
Minimum Value	−0.56	−0.51	−0.47	−0.69	−0.92	−0.76	−0.25	−1.07	1.34	−0.42	−0.82
Maximum Value	0.26	0.27	0.43	0.54	−0.17	0.27	1.36	−0.24	2.76	0.51	0.36
Mean (*N* = 110)	−0.09	−0.04	−0.04	−0.07	−0.49	−0.14	0.55	−0.61	2.09	−0.01	−0.21
S.D. (*N* = 110)	0.25	0.24	0.30	0.37	0.27	0.33	0.42	0.26	0.36	0.28	0.36
**Winter (November)**											
ZY-1	−0.18	0.06	−0.27	−0.03	−0.18	0.11	0.78	−0.32	1.25	−0.20	0.05
ZY-2	−0.17	0.02	−0.30	0.09	−0.30	0.27	0.87	−0.41	1.64	−0.16	0.43
ZY-3	−0.02	−0.31	0.48	−0.41	−0.62	−0.46	0.73	−0.73	1.36	−0.01	−0.28
CT-1	−0.10	0.15	0.15	0.11	−0.2	0.13	0.73	−0.34	1.15	−0.08	0.22
CT-2	0.14	0.33	0.18	0.22	−0.12	0.29	0.73	−0.20	1.29	−0.11	0.33
CT-3	−0.01	0.16	−0.13	0.08	−0.13	0.19	0.86	−0.13	1.58	−0.20	0.38
Minimum Value	−0.18	−0.31	−0.30	−0.41	−0.62	−0.46	0.73	−0.73	1.15	−0.20	−0.28
Maximum Value	0.14	0.33	0.48	0.22	−0.12	0.29	0.87	−0.13	1.64	−0.01	0.43
Mean (N = 42)	−0.05	0.07	0.02	0.01	−0.26	0.09	0.78	−0.35	1.38	−0.13	0.19
S.D. (N = 42)	0.12	0.21	0.3	0.22	0.19	0.28	0.07	0.21	0.19	0.07	0.26

**Table 3:** EF values of seldom monitored trace elements in surface sediments of the Three Gorges Reservoir during summer (July) and winter (November).

Sampling sites	Li	Be	B	V	Ni	Ga	Sn	Sb	Tl	Bi
**Summer (July)**										
ZY-1	1.98	1.29	2.41	1.14	1.08	1.54	0.90	7.34	1.44	1.13
ZY-2	1.56	1.43	1.75	1.25	1.27	1.64	0.90	4.71	1.22	1.20
ZY-3	1.03	1.24	0.89	1.25	1.23	1.82	0.86	4.86	1.12	1.37
MX-1	1.36	1.49	1.52	1.24	1.24	2.11	0.88	8.98	1.37	1.04
MX-2	1.66	1.58	1.61	1.50	1.38	2.20	1.06	6.74	1.76	1.23
MX-3	1.30	1.37	1.00	1.60	1.37	2.64	0.94	6.28	1.60	1.04
MX-4	1.37	1.38	1.38	1.28	1.31	1.98	0.92	6.18	1.42	1.10
MX-5	1.17	1.22	1.14	1.16	1.23	1.88	0.88	8.37	1.18	1.41
CJ	1.00	1.12	0.95	1.18	1.26	1.87	0.80	6.61	1.12	1.17
CT-1	1.28	1.59	2.30	1.37	1.27	2.25	1.02	4.78	1.65	1.28
CT-2	1.20	1.35	1.05	1.64	1.36	2.89	0.95	4.22	1.60	1.05
CT-3	1.15	1.44	1.06	1.39	1.33	1.95	0.93	4.56	1.21	1.54
Minimum Value	1.00	1.12	0.89	1.14	1.08	1.54	0.80	4.22	1.12	1.04
Maximum Value	1.98	1.59	2.41	1.64	1.38	2.89	1.06	8.98	1.76	1.54
Mean (*N* = 110)	1.34	1.37	1.42	1.33	1.28	2.06	0.92	6.14	1.39	1.21
S.D. (*N* = 110)	0.28	0.14	0.52	0.17	0.08	0.39	0.07	1.56	0.23	0.16
**Winter (November)**										
ZY-1	1.00	1.18	0.94	1.11	1.22	1.95	0.91	2.69	0.99	1.17
ZY-2	1.09	1.25	1.00	1.31	1.48	2.24	0.93	3.83	1.10	1.65
ZY-3	1.52	1.24	2.14	1.16	1.11	2.54	0.93	3.95	1.52	1.27
CT-1	1.08	1.28	1.28	1.25	1.26	1.91	0.91	2.56	1.09	1.34
CT-2	1.20	1.36	1.23	1.27	1.33	1.80	0.95	2.65	1.01	1.37
CT-3	1.09	1.22	1.00	1.15	1.25	1.99	1.00	3.26	0.95	1.42
Minimum Value	1.00	1.18	0.94	1.11	1.11	1.80	0.91	2.56	0.95	1.17
Maximum Value	1.52	1.36	2.14	1.31	1.48	2.54	1.00	3.95	1.52	1.65
Mean (N = 42)	1.16	1.26	1.27	1.21	1.27	2.07	0.94	3.16	1.11	1.37
S.D. (N = 42)	0.18	0.06	0.45	0.08	0.12	0.27	0.03	0.62	0.21	0.16

**Table 4:** Rotated component matrix for data of sediments (PCA loadings >0.5 were shown in bold).

	PC1	PC2	PC3	PC4
Li	0.322	**0.660**	0.391	0.190
Be	**0.664**	**0.512**	0.179	−0.197
B	−0.559	0.351	**0.617**	0.096
V	**0.852**	0.473	−0.138	−0.01
Co	0.103	**0.938**	−0.022	0.071
Ni	**0.952**	0.023	−0.028	0.016
Ga	**0.860**	**0.540**	−0.312	0.062
Sn	**0.831**	0.211	0.163	−0.09
Sb	−0.119	−0.022	−0.008	**0.940**
Tl	0.426	**0.786**	−0.069	0.013
Bi	**0.749**	−0.381	0.400	0.009
Cr	**0.927**	0.251	−0.112	−0.058
Mn	**0.734**	−0.248	−0.377	0.190
Cu	**0.819**	−0.288	0.287	−0.002
Zn	**0.876**	−0.339	0.054	−0.008
As	**0.912**	−0.179	0.092	0.083
Cd	**0.817**	−0.283	−0.222	0.016
Pb	**0.667**	−0.584	0.322	−0.035
TOC (%)	−0.352	−0.107	−0.129	−0.114
Clay (%)	**0.771**	−0.299	−0.297	0.202
Percentage of variance	55.517	70.747	76.748	82.253
Cumulative percent	55.517	15.230	6.001	5.505
