# Supplementary material for: The spatial distribution, accumulation and potential source of seldom monitored trace elements in sediments of Three Gorges Reservoir, China
Source: Sci Rep. 2015 Nov 5;5:16170. doi: 10.1038/srep16170 (PMC4633632; doi:10.1038/srep16170)
Supplement: Supporting Information [file srep16170-s1.doc]

**The spatial distribution, accumulation and potential source of seldom monitored trace elements in sediments of Three Gorges Reservoir, China**

Lanfang Han^1, 3^, Bo Gao ^1, 2^*, Huaidong Zhou ^1, 2^, Dongyu Xu ^2^, Xin Wei ^3^, Li Gao ^3^

*^1^State Key Laboratory of Simulation and Regulation of Water Cycle in River Basin,* *China Institute of Water Resources and Hydropower Research, Beijing 100038, China*

^2^*Department of Water Environment, China Institute of Water Resources and Hydropower Research, Beijing, 100038, China*

*^3^State Key Laboratory of Water Environment Simulation, School of Environment, Beijing Normal University, Beijing 100875, China*

**Table numbers: 4**

**Figure numbers: 5**

*Corresponding author. Tel: 86-10-68781891; Fax: 86-10-68781883; E-mail: gaosky34@hotmail.com (B. Gao).

**Figure S1. Grain size distribution of sediments collected from Three Gorge Reservoir in the summer (a) and winter (b)**


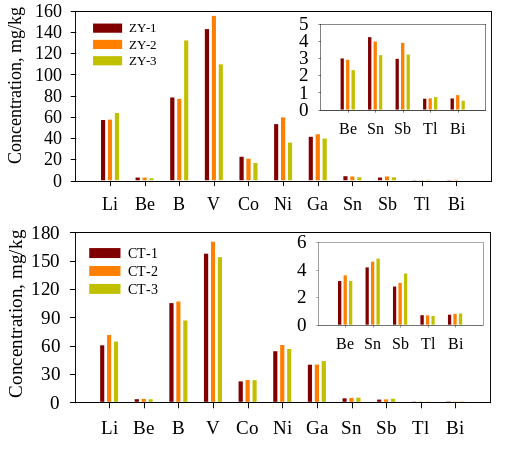


**a**

**b**

**Figure S2. The distribution of concentrations of seldom monitored trace elements in ZY (a) and CT River (b) of the Three Gorge Reservoir during the winter.**

**Figure S3. The distribution of concentrations of Be, Co, Sn, Sb, Tl and Bi with the sediment profiles in the mainstream (CJ) and tributaries (ZY, MX and CT) of the Three Gorge Reservoir during the summer.** Note: a = ZY-1, b = ZY-2, c = ZY-3, d = MX-1, e = MX-2, f = MX-3, g = MX-4, h = MX-5, I = CJ, j = CT-1, k = CT-2, l = CT-3.

**Figure S4. The distribution of concentrations of Li, B, V, Co, Ni and Ga with the sediment profiles in the tributaries (ZY and CT) of the Three Gorge Reservoir during the winter.** Note: a = ZY-1, b = ZY-2, c = ZY-3, d = CT-1, e = CT-2, f = CT-3.

**Figure S5. The distribution of concentrations of Be, Sn, Sb, Tl and Bi with the sediment profiles in the tributaries (ZY and CT) of the Three Gorge Reservoir during the winter.** Note: a = ZY-1, b = ZY-2, c = ZY-3, d = CT-1, e = CT-2, f = CT-3.

| **Table S1. Correlation coefﬁcients of seldom monitored trace elements in sediments of Three Gorge Reservoir during the summer** | | | | | | | | | | | |
| --- | --- | --- | --- | --- | --- | --- | --- | --- | --- | --- | --- |
|  | Li | Be | B | V | Co | Ni | Ga | Sn | Sb | Tl | Bi |
| Li | 1 |  |  |  |  |  |  |  |  |  |  |
| Be | 0.485^b^ | 1 |  |  |  |  |  |  |  |  |  |
| B | 0.410^b^ | 0.011 | 1 |  |  |  |  |  |  |  |  |
| V | 0.483^b^ | 0.735^b^ | -0.350^b^ | 1 |  |  |  |  |  |  |  |
| Co | 0.283^b^ | 0.447^b^ | -0.540^b^ | 0.668^b^ | 1 |  |  |  |  |  |  |
| Ni | 0.327^b^ | 0.540^b^ | -0.510^b^ | 0.793^b^ | 0.955^b^ | 1 |  |  |  |  |  |
| Ga | 0.448^b^ | 0.538^b^ | -0.314^b^ | 0.865^b^ | 0.643^b^ | 0.705^b^ | 1 |  |  |  |  |
| Sn | 0.397^b^ | 0.782^b^ | -0.220^a^ | 0.883^b^ | 0.729^b^ | 0.747^b^ | 0.586^b^ | 1 |  |  |  |
| Sb | 0.112 | 0.067 | -0.213^a^ | 0.220^a^ | 0.336^b^ | 0.340^b^ | 0.283^b^ | 0.313^b^ | 1 |  |  |
| Tl | 0.604^b^ | 0.676^b^ | 0.029 | 0.786^b^ | 0.377^b^ | 0.492^b^ | 0.818^b^ | 0.623^b^ | 0.198^a^ | 1 |  |
| Bi | 0.159 | 0.363^b^ | -0.256^b^ | 0.430^b^ | 0.729^b^ | 0.651^b^ | 0.185 | 0.678^b^ | 0.312^b^ | 0.065 | 1 |
| Cr | 0.384^b^ | 0.692^b^ | -0.464^b^ | 0.927^b^ | 0.485^b^ | 0.926^b^ | 0.798^b^ | 0.828^b^ | 0.366^b^ | 0.656^b^ | 0.520^b^ |
| Mn | 0.054 | 0.214^a^ | -0.690^b^ | 0.668^b^ | 0.889^b^ | 0.831^b^ | 0.593^b^ | 0.535^b^ | 0.258^b^ | 0.180 | 0.580^b^ |
| Cu | 0.128 | 0.325^b^ | -0.340^b^ | 0.552^b^ | 0.804^b^ | 0.766^b^ | 0.362^b^ | 0.609^b^ | 0.386^b^ | 0.138 | 0.761^b^ |
| Zn | 0.131 | 0.395^b^ | -0.515^b^ | 0.620^b^ | 0.861^b^ | 0.799^b^ | 0.460^b^ | 0.751^b^ | 0.398^b^ | 0.196^a^ | 0.813^b^ |
| As | 0.237^b^ | 0.415^b^ | -0.459^b^ | 0.693^b^ | 0.907^b^ | 0.860^b^ | 0.502^b^ | 0.704^b^ | 0.309^b^ | 0.231^a^ | 0.777^b^ |
| Cd | -0.005 | 0.228^a^ | -0.609^b^ | 0.561^b^ | 0.781^b^ | 0.703^b^ | 0.456^b^ | 0.639^b^ | 0.311^b^ | 0.123 | 0.646^b^ |
| Pb | -0.074 | 0.220^a^ | -0.320^b^ | 0.249 ^b^ | 0.605^b^ | 0.516^b^ | 0.071 | 0.563^b^ | 0.394^b^ | -0.122 | 0.785^b^ |
| TOC (%) | -0.222^a^ | -0.109 | 0.139 | -0.186 | -0.215^a^ | -0.206^a^ | -0.143 | -0.136 | -0.092 | -0.042 | -0.134 |
| Clay (%) | 0.388^a^ | 0.333^a^ | 0.500^b^ | 0.468^b^ | 0.707^b^ | 0.801^b^ | 0.533^b^ | 0.501^b^ | 0.326^b^ | 0.500^b^ | 0.620^b^ |
| ^a^ Correlation is signiﬁcant at the 0.05 level (two-tailed); ^b^ Correlation is signiﬁcant at the 0.01 level (two-tailed). | | | | | | | | | | | |

| **Table S2. Correlation coefﬁcients of seldom monitored trace elements in sediments of Three Gorge Reservoir during the winter** | | | | | | | | | | | |
| --- | --- | --- | --- | --- | --- | --- | --- | --- | --- | --- | --- |
|  | Li | Be | B | V | Co | Ni | Ga | Sn | Sb | Tl | Bi |
| Li | 1 |  |  |  |  |  |  |  |  |  |  |
| Be | 0.484^b^ | 1 |  |  |  |  |  |  |  |  |  |
| B | 0.001 | -0.543^b^ | 1 |  |  |  |  |  |  |  |  |
| V | 0.609^b^ | 0.922^b^ | -0.576^b^ | 1 |  |  |  |  |  |  |  |
| Co | 0.272 | 0.562^b^ | -0.706^b^ | 0.579^b^ | 1 |  |  |  |  |  |  |
| Ni | 0.338^a^ | 0.703^b^ | -0.745^b^ | 0.760^b^ | 0.899^b^ | 1 |  |  |  |  |  |
| Ga | 0.660^b^ | 0.912^b^ | -0.419^b^ | 0.887^b^ | 0.385^a^ | 0.579^b^ | 1 |  |  |  |  |
| Sn | 0.436 | -0.26 | -0.617^b^ | 0.895^b^ | 0.733^b^ | 0.814^b^ | 0.696^b^ | 1 |  |  |  |
| Sb | -0.004 | 0.639^b^ | 0.254 | -0.198 | -0.191 | -0.214 | -0.16 | -0.238 | 1 |  |  |
| Tl | 0.585^b^ | 0.349^a^ | 0.067 | 0.743^b^ | -0.05 | 0.324^a^ | 0.802^b^ | 0.562^b^ | -0.085 | 1 |  |
| Bi | 0.038 | 0.877^b^ | -0.527^b^ | 0.920^b^ | 0.807^b^ | 0.832^b^ | 0.147 | 0.567^b^ | -0.149 | -0.006 | 1 |
| Cr | 0.472^b^ | 0.222 | -0.749^b^ | 0.233 | 0.788^b^ | 0.910^b^ | 0.767^b^ | 0.937^b^ | -0.259 | 0.535^b^ | 0.605^b^ |
| Mn | 0.266 | 0.520^b^ | -0.437^b^ | 0.554^b^ | 0.482^b^ | 0.413^b^ | 0.268 | 0.315^a^ | -0.091 | 0.007 | 0.406^b^ |
| Cu | 0.133 | 0.48 | -0.605^b^ | 0.457^b^ | 0.885^b^ | 0.915^b^ | 0.333^a^ | 0.697^b^ | -0.18 | 0.14 | 0.920^b^ |
| Zn | 0.117 | 0.644^b^ | -0.739^b^ | 0.691^b^ | 0.881^b^ | 0.759^b^ | 0.251 | 0.720^b^ | -0.194 | -0.056 | 0.744^b^ |
| As | 0.350^a^ | 0.630^b^ | -0.702^b^ | 0.629^b^ | 0.899^b^ | 0.934^b^ | 0.490^b^ | 0.755^b^ | -0.173 | 0.205 | 0.820^b^ |
| Cd | 0.331^a^ | 0.251 | -0.755^b^ | 0.209 | 0.778^b^ | 0.700^b^ | 0.503^b^ | 0.749^b^ | -0.196 | 0.149 | 0.491^b^ |
| Pb | -0.104 | 0.349^a^ | -0.577^b^ | 0.743^b^ | 0.807^b^ | 0.654^b^ | -0.016 | 0.519^b^ | -0.147 | -0.235 | 0.835^b^ |
| TOC (%) | -0.409^b^ | -0.627^b^ | -0.455^b^ | -0.679^b^ | -0.376^b^ | -0.536^b^ | -0.521^b^ | -0.612^b^ | 0.047 | -0.410^b^ | -0.325 |
| Clay (%) | 0.206 | 0.501^b^ | -0.424^b^ | 0.511^b^ | 0.452^b^ | 0.444^b^ | 0.241 | 0.410^a^ | -0.091 | 0.041 | 0.448^b^ |
| ^a^ Correlation is signiﬁcant at the 0.05 level (two-tailed); ^b^ Correlation is signiﬁcant at the 0.01 level (two-tailed). | | | | | | | | | | | |

| **Table S3. Means, ranges and standard deviation (S.D.) of major element contents in sediments of Three Gorge Reservoir (mg/kg)** | | | | | | | |
| --- | --- | --- | --- | --- | --- | --- | --- |
|  | Cr | Mn | Cu | Zn | As | Cd | Pb |
| Sampling period at summer (July) | | | | | | | |
| Minimum Value | 51.74 | 218.23 | 22.93 | 64.8 | 7.35 | 0.17 | 19.03 |
| Maximum Value | 129.23 | 766.71 | 98.86 | 270.34 | 20.4 | 2.52 | 121.73 |
| Mean (*N* = 110 ) | 101.56 | 504.03 | 57.11 | 146.04 | 14.69 | 0.86 | 43.52 |
| S.D. (*N* = 110 ) | 15 | 164.07 | 15.29 | 37.86 | 3.23 | 0.47 | 15.96 |
| Sampling period at Winter (November) | | | | | | | |
| Minimum Value | 68.44 | 412.24 | 30.4 | 78.39 | 9.31 | 0.2 | 19.48 |
| Maximum Value | 131.08 | 716.37 | 124.44 | 271.24 | 20.82 | 2.14 | 99.88 |
| Mean (N = 42 ) | 99.82 | 580.35 | 60.01 | 139.89 | 14.55 | 0.82 | 42.37 |
| S.D. (N = 42 ) | 18.18 | 70.87 | 22.99 | 45.19 | 3.28 | 0.47 | 20.47 |

| **Table S4. GSD-12 certified values, analytical values and recovery** | | | |
| --- | --- | --- | --- |
| Element | Analytical value (mg/kg) | Certified values (mg/kg) | Recovery (%) |
| Li | 12.78 | 13.0 | 98.3 |
| Be | 0.76 | 0.90 | 86.7 |
| Bi | 0.35 | 0.38 | 92.1 |
| B | 27.18 | 26.00 | 104.5 |
| V | 104.75 | 107 | 97.9 |
| Co | 15.01 | 15.3 | 98.1 |
| Ni | 12.5 | 12.8 | 97.7 |
| Ga | 7.05 | 6.4 | 110.2 |
| Sn | 1.24 | 1.4 | 88.6 |
| Sb | 6.45 | 6.3 | 102.4 |
| Tl | 0.20 | 0.21 | 95.2 |
